# A semi-nonparametric mixture model for selecting functionally consistent proteins

**DOI:** 10.1186/1471-2105-11-486

**Published:** 2010-09-28

**Authors:** Lianbo Yu, RW Doerge

**Affiliations:** 1 Center for Biostatistics, The Ohio State University, Columbus, OH 43221, USA; 2 Department of Statistics, Purdue University, West Lafayette, IN 47907, USA

## Abstract

**Background:**

High-throughput technologies have led to a new era of proteomics. Although protein microarray experiments are becoming more common place there are a variety of experimental and statistical issues that have yet to be addressed, and that will carry over to new high-throughput technologies unless they are investigated. One of the largest of these challenges is the selection of functionally consistent proteins.

**Results:**

We present a novel semi-nonparametric mixture model for classifying proteins as consistent or inconsistent while controlling the false discovery rate and the false non-discovery rate. The performance of the proposed approach is compared to current methods via simulation under a variety of experimental conditions.

**Conclusions:**

We provide a statistical method for selecting functionally consistent proteins in the context of protein microarray experiments, but the proposed semi-nonparametric mixture model method can certainly be generalized to solve other mixture data problems. The main advantage of this approach is that it provides the posterior probability of consistency for each protein.

## Background

Over the last decade or longer, microarray technology has been used for measuring gene expression and has greatly impacted biomarker discovery [1], transcription factor identification [2], the assessment of gene interactions [3], and the detection of biological pathways [4]. Despite the massive application of microarrays to transcriptome applications there are limitations to the extent of the conclusions that can be made. Messenger RNA (mRNA) is the intermediate product of genes, with proteins being the final products and the key factors of metabolism. Although the levels of mRNA and protein for a gene are related they are not always highly correlated, which can be due to many reasons, e.g., translation rate, protein stability, and post-translational modification, etc. [5]. Given that the motivation and goal of many experiments is to understand not only the function of genes, but the network of genes that encode proteins, the abundance of proteins themselves are of increasing interest. Toward this end, microarray technology when adapted to proteins, are known as protein microarrays, and have been developed and widely used to assess the abundance of proteins [6-12]. The similarities between microarray technology as applied to gene expression [13], and as applied to protein abundance, are the same in that improved accuracy and precision, as well as design issues and normalization techniques for protein microarrays have been established [14,15].

Screening and identifying proteins as potential medical diagnostics and disease classification biomarkers is the main motivation of many protein microarray experiments [16-21]. The precursor to any successful screening application, and an essential issue that must be resolved to ensure that the accurate protein abundance measurements can be obtained by protein microarrays, is the consistency of a protein to report hybridization abundance. The protein itself is the probe on the array, and since proteins have a complex three dimensional structure, the structure itself, as well as the orientation of a protein, need to be retained. Toward this end, it is highly unlikely that every protein will be functional since different proteins often require different environment conditions for maintaining structures, and are typically much less stable than DNA. If the three dimensional nature of the structure is lost, or the required functional portion of the protein is not available to bind its target protein (i.e., the sample), the target protein abundance measurement will be much smaller than it should be, or missed all together. Proteins whose structure or function are not maintained when attached to the array as probes are called inconsistent proteins, and if used provide inflated biomarker error rates (i.e., false positive rate and false negative rate). Alternatively, proteins that retain their structure and function are called consistent proteins and are desirable as probes on the array, and ultimately potential biomarkers. As such the selection of proteins that maintain functional consistency across experiments is a major and necessary requirement in the design and analysis of protein microarray experiments [17].

Certainly, high-throughput chemical validation of protein consistency is possible, but it is expensive and time consuming. Toward this end it is possible to statistically estimate protein consistency. In its simplest form, Pearson's correlation coefficient has been employed as a consistency measure in an antibody microarray study by Miller *et al*. [17], but it only measures the linearity of repeated measurements, and therefore is limited in its usefulness. A concordance correlation coefficient that is able to measure the consistency of repeated measurements was proposed by Lin [22], and later expanded to a total deviation index (TDI) [23], which provides a boundary within which a certain required percentage of differences between paired observations is obtained while controlling the error rate. As described by Lin [24], TDI and the concordance correlation coefficient provide the same information, but from different perspectives, and thus share their limitations. Namely, both the concordance correlation coefficient and TDI only demonstrate good asymptotic properties under the assumption of normality; a reality that is often questionable in application. Furthermore, the comparability of concordance correlation coefficients across proteins requires the ranges of the abundance measurements of proteins to be similar, which is not practical in large scale experiments [25]. To address the challenges and issues that are associated with identifying functionally consistent proteins, we propose a new statistic based on variance components from an analysis of variance (ANOVA) model. We rely on a mixture model to achieve this goal. Applications of mixture models in biology have proven to be excellent for separating data into the correct number of classes. For example, Efron *et al*. [26] proposed a two-component mixture model for testing differential expression. In this application the distributions of the t-statistics from both differentially expressed genes and non-differentially expressed genes were estimated by a nonparametric method, but the tail probabilities were not able to be estimated accurately. Toward this end the accuracy of estimating the tail probability was improved by using a two-component mixture model Pan *et al*. [27] where a finite normal mixture was assumed for each component. For microarray data it certainly is possible to simulate test statistics under the null hypothesis (i.e., a single component) using permutation theory since the treatment conditions for testing differences are known. However, for protein array data the first challenge is to identify proteins that are consistent, and then work only with these data. In other words, we are focusing on separating proteins into inconsistent and consistent classes, and then using only the informative proteins (i.e., consistent proteins) to address the biological question(s). To achieve this we propose a novel two-component semi-nonparametric mixture model. Simulations demonstrate the performance of the proposed approach and provide food for thought when designing future protein microarray experiments. We also apply the proposed approach to real data for the purpose of demonstrating its usefulness.

## Results and Discussion

Simulations were conducted for the purpose of providing insight into the performance and value of the proposed semi-nonparametric approach. Data were simulated from known consistency classifications. Data were analysed with the proposed approach and the number of times proteins are correctly classified is recorded. From these simulation results, false discovery rate, as well as false non-discovery rate were calculated and are discussed.

### A power study

Simulations were designed to study the statistical power of the approach under different sample sizes and different underlying two-component mixture distributions. Data were simulated directly from nine unique two-component semi-nonparametric mixture distributions with specified parameter values (Table 1). The tuning parameter *K *took on values 0, 1, or 2 for each semi-nonparametric density in each mixture. Sample sizes are 50, 100, 300, or 500. The proportions of the first mixture component with smaller mean, *λ*_0_, are 0.20, 0.50, or 0.80. The distance between the two mixture components are 1 or 2, where the distance is defined as

**Table 1:** Nine different simulation scenarios.

		Component 1				Component 2			
			
model	distance	***ϕ***_**11**_	***ϕ***_**12**_	***µ***_**1**_	***σ***_**1**_	***ϕ***_**21**_	***ϕ***_**22**_	***µ***_**2**_	***σ***_**2**_
1	D = 1	*π*/2	*π*/2	12	2	*π*/2	*π*/2	17	3
	D = 2	*π*/2	*π*/2	12	2	*π*/2	*π*/2	24	4

2	D = 1	*π*/2	*π*/2	12	2	2.17	*π*/2	19.4	3
	D = 2	*π*/2	*π*/2	12	2	2.17	*π*/2	26.7	4

3	D = 1	*π*/2	*π*/2	12	2	2	2.75	18	2
	D = 2	*π*/2	*π*/2	12	2	2	2.75	30.3	4

4	D = 1	0.97	*π*/2	9.8	2.3	*π*/2	*π*/2	17	3
	D = 2	0.97	*π*/2	9.8	2.3	*π*/2	*π*/2	24	4

5	D = 1	0.97	*π*/2	10	2	2.17	*π*/2	18.15	2.5
	D = 2	0.97	*π*/2	10	2	2.17	*π*/2	26.15	4

6	D = 1	0.97	*π*/2	9.8	2.3	2	2.75	19.8	2.5
	D = 2	0.97	*π*/2	9.8	2.3	2	2.75	31.3	4

7	D = 1	4.1	0.9	9.7	1.8	*π*/2	*π*/2	17	3
	D = 2	4.1	0.9	9.7	1.8	*π*/2	*π*/2	24	4

8	D = 1	4.1	0.9	9.7	1.8	2.17	*π*/2	20.5	3.5
	D = 2	4.1	0.9	9.7	1.8	2.17	*π*/2	28.5	4.6

9	D = 1	4.1	0.9	10	2	2	2.75	19.8	2.5
	D = 2	4.1	0.9	10	2	2	2.75	31.3	4

Each simulation model is based on a two-component semi-nonparametric (SNP) mixture distribution that has eight parameters: *ϕ*_11_, *ϕ*_12_, *μ*_1_, *σ*_1_, *ϕ*_21_, *ϕ*_22_, *μ*_2_, *σ*_2_. The distance D of two components is 1 or 2.

(1) $$D=\frac{\left|\mu_{1}-\mu_{2}\right|}{\sigma_{1}+\sigma_{2}},$$

and *μ*_1 _and *μ*_2 _represent the means of two components respectively, while *σ*_1 _and *σ*_2 _represent the standard deviations of two components, respectively. Under each combination of model settings, 1000 data sets were generated.

**Figure 1:**
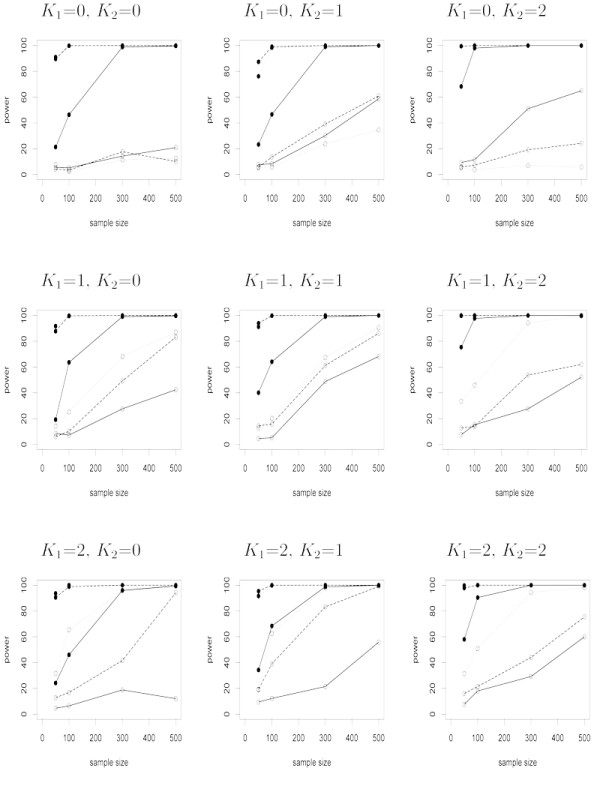
**Power results for nine simulation settings. Schwartz Bayesian Information Criterion (BIC) provides the model selection criterion**. Data were simulated under nine semi-nonparametric (SNP) mixture distributions with the tuning parameter K taking values 0, 1, or 2 for each SNP density. Sample sizes are 50, 100, 300, or 500. *λ*_0 _is 0.2, 0.5, or 0.8. The distance between the means of the component distributions is *D *and has values of 1 or 2. Power was calculated as the proportion of correctly rejected hypothesis for 1000 simulated data sets. Solid curves represent *λ*_0 _= 0.20 and *D *= 1(*○*) or *D *= 2(●). Dashed curves represent *λ*_0 _= 0.50 and *D *= 1(*○*) or *D *= 2(●). Dotted curves represent *λ*_0 _= 0.80 and *D *= 1(*○*) or *D *= 2(●).

For each simulated data scenario, a two-component mixture model was fit to the data. The Expectation-maximization (EM) quasi-Newton algorithm was employed to estimate the model parameters. Model selection criteria Akaike's Information Criterion (AIC) [28], Schwartz Bayesian Information Criterion (BIC) [29] and Hannan-Quinn Criterion (HQ) [30] were used to select the best model. A likelihood ratio test (19; see Methods) was employed to determine whether the mixture distribution was identifiable as two-components (18). A bootstrap method approach approximated the null distribution of the likelihood ratio test statistics, and provided a significance threshold for the likelihood ratio test statistic (see Methods). Power was calculated by estimating the proportion of correctly rejected hypotheses for each of 1000 data sets. Power comparisons for all parameter settings using BIC model selection criteria are provided in Figures 1. The general trend across all three model selection criteria is that well separated mixture distributions (*D *= 2) outperform mixtures that are not well identified (*D *= 1). When the mixtures are well defined, there is obvious increased power for situations where the mixing proportion (*λ*_0_) of the functionally consistent component of the mixture distribution is 50% or greater. Recall, when the tuning parameters *K*_1 _and *K*_2 _are both zero the semi-nonparametric densities in the mixture distribution are both standard normal densities.

As expected, higher power is associated with larger sample size. Dramatically higher power is achieved when the distance between the two components is increased from 1 to 2 simply because the null hypothesis (18) is easier to reject when the mixture components are well separated. Furthermore, AIC tends to choose a larger model that has a larger likelihood ratio test statistic (19) when compared to the smaller model chosen by BIC or HQ [31], therefore the use of AIC yields higher power than BIC or HQ.

### Simulated Data Scenario

The performance of the proposed mixture model with semi-nonparametric densities is evaluated for selecting functionally consistent proteins in a simulation setting based on a real experiment. Since we are interested in understanding the performance of the proposed approach it is necessary to rely on simulated data, rather than actual data since the truth for real data is unknown. Protein microarray data were simulated based on the data scenario described in Zhou *et al*. [32]. Specifically, there are three groups of patients with different stages of disease, and one group of healthy patients. Each group consists of 10 patients (40 patients total). For each patient, hybridization abundance was measured on 300 proteins. Each of the 300 proteins was represented as a probe on the array. Onboard probe (technical) replication allowed each protein to be represented 6 times on the array. Forty samples were individually mixed with a reference sample, hybridized to an array, and the entire experiment was repeated twice. Protein microarray data were simulated as follows

(2) $$log_{2}(\mu_{j\;k})=\mu +G_{j}+S_{k(j)},$$

where *μ_jk _*represents hybridization abundance of individual or patient *k *in group *j*, *μ *represents the overall mean abundance, *G_j _*represents the fixed effect of group *j*, *S_k(j) _*represents the random effect of patient *k *in group *j *following a normal distribution with mean 0 and variance $\sigma_{Sj}^{2}$, and

(3) $$\begin{array}{c} y_{ijkl}=\theta_{jk}+\delta_{ijk}+\epsilon_{ijkl}\\ =log_{2}(\mu_{jk})-log_{2}(\bar{\mu }..)+\delta_{ijk}+\epsilon_{ijkl}\\ =C+G_{j}+S_{k(j)}+\delta_{ijk}+\epsilon_{ijkl}, \end{array}$$

where $C=-log_{2}\frac{\sum_{j,k}2^{G_{j}+{\text{S}}_{k(j)}}}{40}$, *i *= 1,2, *j *= 1,2,3,4, *k *= 1,2, ⋯, 10, *l *= 1,2, ⋯, 6. *y_ijkl _*represents the *l*th log signal ratio of patient *k *to the reference sample in group *j *for experiment i, *θ_jk _*represents the mean log signal ratio of the patient k sample to the reference in group *j*, $\bar{\mu }..$ represents the average of *μ_jk_*'s over *j *and *k*, *δ_ijk _*represents the random error of experiment *i *for patient *k *in group *j*, and ϵ*_ijkl _*represents the *l*th random error within experiment *i *for patient *k *in group *j*. Assume that *δ_ijk _*is from a normal distribution with mean zero and variance $\sigma_{\delta }^{2}$, ϵ*_ijkl _*is from a normal distribution with mean zero and variance $\sigma_{e}^{2}$.

The model parameter settings for the simulation were taken from the aforementioned Zhou et al. antibody microarray data [32], such that the ${G^{'}}_{j}\text{s}$ were sampled from uniform distribution *U*[-1, 1], $\sigma_{Sj}^{2}$ = (0.4*v*)^2^, where *v *were sampled from *U*[0.5, 2] for different *j*, $\sigma_{\delta }^{2}$ = 0.2^2^, and $\sigma_{e}^{2}$ = 0.15^2^. The hybridization abundance data for 300 functionally consistent proteins on each array were simulated from model (2) and (3) (see Methods). Fifty percent of these simulated proteins were randomly chosen to be functionally inconsistent proteins by adding a random between-array deviation with mean 0 and standard deviation drawn from *U *[0.05, 0.5], as well as a random within-array deviation with mean 0 and standard deviation taken from *U*[0.1, 0.4], to a randomly chosen number of separate arrays. Protein classification resulted from estimating the variance components in the ANOVA model (4; see Methods), and modelling the between and within-array variance component statistic with a semi-nonparametric mixture model. The main advantage of the proposed mixture model approach is that it provides the posterior probability of consistency for each protein which in turn establishes the classification rule, as well as estimates the respective error rates.

**Figure 2:**
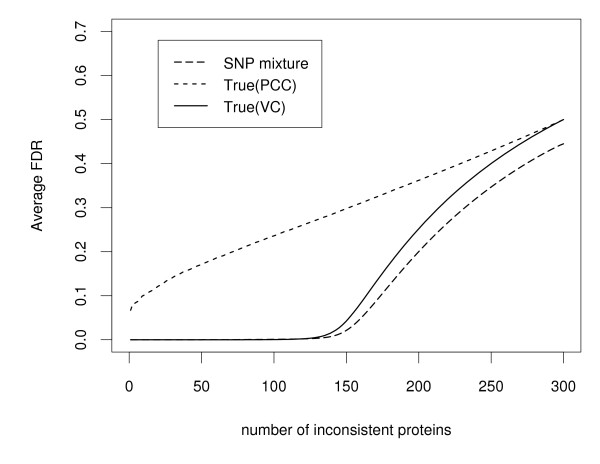
**Comparison of false discovery rates (FDR) using simulated data based on 40 patients and 6 onboard probe replicates**. False discovery rates are averaged over 1000 simulations. Solid curves are the true false discovery rates based on the between- and within-array variance component (VC) statistic, short-dashed curves are the true false discovery rates based on Pearson's correlation coefficient (PCC), and long-dashed curves are the estimated false discovery rates based on the proposed semi-nonparametric (SNP) mixture model method.

**Figure 3:**
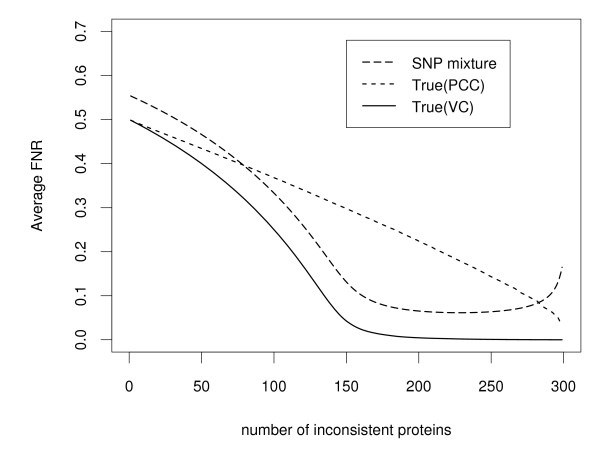
**Comparison of false non-discovery rates (FNR) for the simulated data with 40 patients and 6 onboard probe replicates**. False non-discovery rates are averaged over 1000 simulations. Solid curves are the true false non-discovery rates based on the between- and within-array variance component (VC) statistic, short-dashed curves are the true false non-discovery rates based on Pearson's correlation coefficient (PCC), and long-dashed curves are the estimated false non-discovery rates based on the proposed semi-nonparametric (SNP) mixture model method.

One thousand data simulations were performed under the same simulation setting, and for each the sum of the between- and within-array variation (see Methods) provides the statistic that is ultimately modelled and used for classifying each of the 300 proteins by fitting to the semi-nonparametric mixture model (5). Posterior probabilities defined in Equation (21) and Equation (22) were computed, and then used to calculate the estimated false discovery rate (FDR) in Equation (24), and the estimated false non-discovery rate (FNR) in Equation (25). Since these are simulated data for which we know the true classification, the true FDR and FNR were calculated and compared to the respective estimated values from the simulated data analyses. The estimated and true FDR and FNR were averaged over 1000 simulations, respectively. The average FDR (Figure 2) and FNR (Figure 3) were plotted against the number of inconsistent proteins. The conservative nature of this approach is illustrated in the downward bias of the FDR estimates and the corresponding upward bias of the FNR estimates.

We compare the proposed semi-nonparametric approach to the work of Miller et al. [17] who selected functionally consistent proteins using an arbitrary cutoff value for Pearson's correlation coefficient. It is important to realize that their cutoff value is not statistically justified, nor does it provide error rate control. We calculated Pearson's correlation coefficients (PCC) for each of the 1000 simulated data sets, and reported in the average FDR and FNR results in Figures 2 and 3, respectively. Not surprisingly, larger true error rates are experienced for the Pearson's correlation coefficient when compared to the variance component (VC) statistic that is based on the between- and within-array variation. Essentially, the variation in the random error(s) captures the difference between consistent and inconsistent proteins allowing the variance estimate based on between- and within-array variation to provide information about protein consistency. Based on this rationale, the misclassification error rates of the proposed approach are expected to be smaller than the Pearson's correlation coefficient. As can be seen for Pearson's correlation coefficient, when the number of inconsistent proteins is 160, the false discovery rate is 0.310 (Figure 2) and the false non-discovery rate is 0.283 (Figure 3). By comparison, based on the between- and within-array variation statistics the false discovery rate is 0.083 and the false non-discovery rate is 0.024. The same phenomena occur at any other number of inconsistent proteins (Figures 2 - 3).

### Biological and technical replication

**Figure 4:**
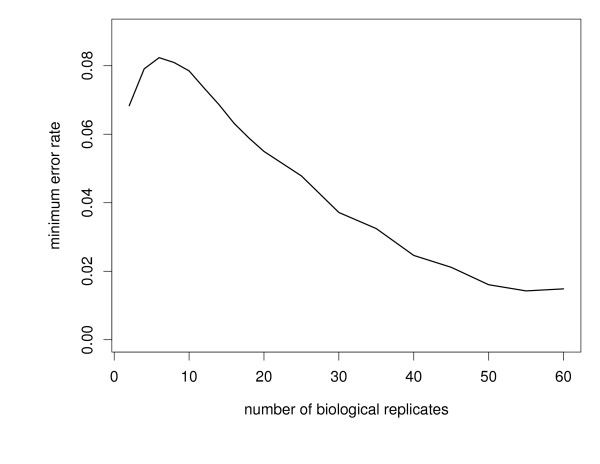
**Minimum classification error rate for increasing numbers of biological replicates (i.e., increasing patient number)**. Minimum classification error rate was computed for each number of replicates ranging from 2 to 60 for a fixed number (6) of protein probes. Larger numbers of replicates/patients achieve greater classification results.

**Figure 5:**
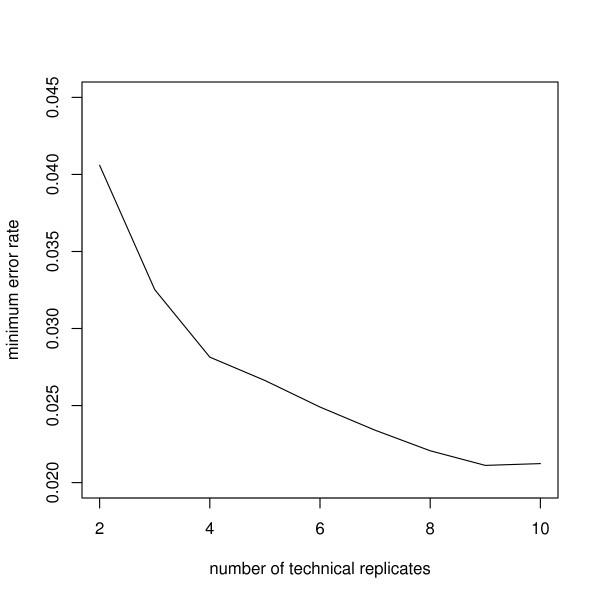
**Minimum classification error rate for increasing numbers of technical replicates (i.e., number of onboard replicates for each protein probe)**. Minimum classification error rate was computed for each number of technical replicates (2 to 10) for a fixed number (40) of biological replicates (or patients). Larger numbers of technical replicates achieve better classification results.

We explored the influence of the number of biological replicates (or, total patient number) and technical replicates (or, onboard per protein probe replicate) on the proposed semi-nonparmetric mixture model approach for selecting functionally consistent proteins using two different simulation settings. Data were simulated from model (2) and (3) (see Methods). The first simulation focused on the number of biological replicates or patients (2 to 60) while fixing the number of onboard probe replicates representing each protein at 6. Six onboard replicates is a relatively large number and is in keeping with many of the current protein microarray investigations. The classification error rate was computed by minimizing (26; see Methods) for each number of replicates (Figure 4) under consideration, and it can be seen that rate drops off quickly as the number of replicates increases from 6 to 50. The second simulation evaluated the number of onboard per protein probe replicates while fixing the number of biological replicates (or patients) at 40. Figure 5 illustrates the classification error rates dropping as the number of onboard replicates increases. Clearly, the largest decrease is most dramatic in the range from 2 to 4.

### A case study

We applied our method to data from an antibody microarray experiment from Zhou *et al*. [32]. Two-color rolling-circle amplification (RCA) was used to assess thirty five antibody proteins from duplicate sets of twenty four serum samples using antibody microarrays prepared on nitrocellulose. The twenty four serum samples consist of six liver cancer patients, six pre-cirrhotic patients, six cirrhotic, and six normals. Each antibody has 5 replicates on the array.

**Figure 6:**
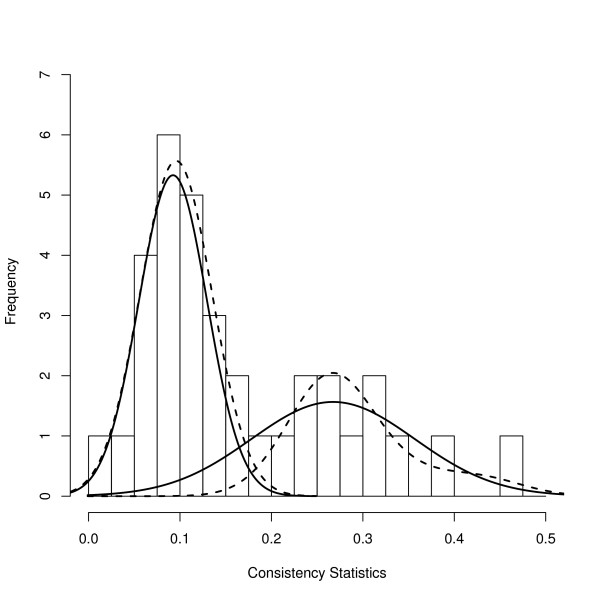
**Histogram of consistent statistics**. Solid curves are the estimated densities where *K *= 0 for both components. These results are based on the BIC model selection criterion. Dashed curves are the estimated densities where *K *= 2 for both components.

**Figure 7:**
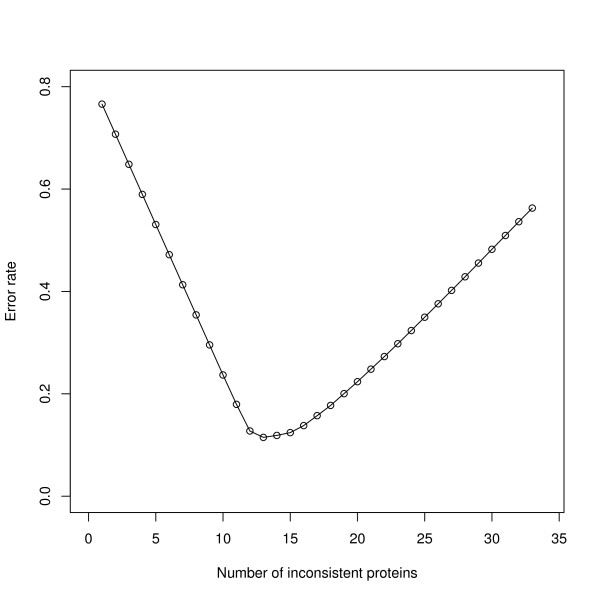
**Error rate for claiming the number of inconsistent proteins**. The estimated classification error rate plotted against each number of potential inconsistent proteins. The estimated FDR (24) and FNR (25) was calculated using a 2:1 ratio for the cost of FDR and FNR. The minimum error rate occurs when 13 proteins are found as inconsistent while 21 protein are consistent.

In the analysis, one antibody was removed due to no signal. The ANOVA model (4) was employed to calculate the total variation due to random error. The range of the consistency statistics is shown in a histogram (Figure 6), from which we can see there are two clusters. To test whether the two components of the mixture model (i.e., consistent and inconsistent proteins) are separable we calculated the likelihood ratio test (19) and found it to be 13.65, for which significance was determined by comparing with a critical value under the null hypothesis. To do that, we bootstrapped the null hypothesis likelihood ratio test statistics and obtained a p-value of 0.022 for the likelihood ratio test statistics that corresponds to the value 13.65, therefore we rejected the null hypothesis and concluded that the consistent and inconsistent proteins are separable. Both components, as determined by the BIC criterion, under the alternative hypothesis have *K *= 0, and have the model parameter estimate ${\hat{\mu }}_{0}$ = 0.092, ${\hat{\sigma }}_{0}$ = 0.037, ${\hat{\mu }}_{1}$ = 0.267, ${\hat{\sigma }}_{1}$ = 0.089 and ${\hat{\lambda }}_{0}$ = 0.588. Because the sample size (number of proteins) is small, model selection tends to choose simpler models where *K *= 0. We illustrate the complex densities where *K *= 2 for both components in Figure 6. In order to determine the optimal number of functionally consistent proteins, we estimated the FDR (24) and FNR (25) and obtained the error rate (26) by using a 2:1 ratio for the cost of FDR and FNR. Figure 7 shows that the minimum error rate occurs when there are 13 inconsistent proteins and 21 consistent proteins. In Zhou *et al*. [32], Pearson's correlation coefficient was calculated for each protein to evaluate measurement reproducibility. Unfortunately, Pearson's correlation coefficient does not provide a classification of consistent and inconsistent proteins, so it is not possible to compare the results of our approach with the published results from Zhou *et al*. [32].

## Discussion

The challenge of selecting and employing functionally consistent proteins for protein microarray experiments is complicated by the three-dimensional structure of the protein itself. Specifically, the proteins that are spotted on to the array as probes (during the fabrication of the array) need to maintain functional consistency for each sample hybridized to the array, as well as across experiments. Identifying and employing functionally consistent proteins continues to be a major and necessary concern in both the design and analysis of protein microarray experiments. To address this concern, a novel statistical approach based on modelling the between- and within-array variation, using a semi-nonparametric mixture model, is presented for the purpose of discriminating functionally consistent proteins. Of course, once functionally consistent proteins have been identified and the array fabricated, it is then necessary to develop additional statistical methods that can detect proteins of differing abundance.

After classifying proteins as consistent and inconsistent proteins, the abundance data from functionally consistent proteins can be used for differential protein abundance/expression analysis. The semi-nonparametric mixture model that was initially proposed to select functionally consistent proteins (5) can also be adapted for detecting differentially expressed proteins. Specifically, one component of the mixture identifies the non-differentially expressed proteins, while the other component acknowledges the differentially expressed proteins. The semi-nonparametric mixture model lies between parametric and nonparametric approaches since it does not put distributional assumption on the data themselves, but on the test statistics. The semi-nonparametric mixture model as applied to differential expression analysis was investigated and shows great performance [33].

The proposed semi-nonparametric mixture model is a novel and broadly applicable approach in the mixture model literature. For applications to either identifying functionally consistent proteins, or testing for differential protein abundance between samples, only two-component mixture models are employed. The extension of the semi-nonparametric mixture model to a multiple-component and multivariate mixture model has potential to address high-dimensional problems for the purpose of classification, and it has potential to work for a variety of data problems since it provides the flexibility necessary for model fitting.

## Conclusions

A novel semi-nonparametric mixture model is proposed for the purpose of selecting functionally consistent proteins that can be used for protein microarray experiments. The proposed approach is able to attach a posterior probability of being inconsistent to each protein, from which false discovery and false non-discovery rates can be estimated. We validated the performance of our method through simulations. Additionally, the characteristics of the semi-nonparametric mixture model were studied by a power analysis. Our novel method provides an improvement in the accuracy of proteins that are selected as probes on a protein microarray, as well as an alternative approach to studying a variety of additional mixture data problems.

## Methods

### ANOVA model

Consider a repeated protein microarray experiment. There are *m *proteins (probes) spotted on *n *arrays. These *n *arrays are used to hybridize material for *n *test samples from *J *different patient groups. The same amount of a reference sample is mixed with each test sample, and each mixture is hybridized on one of *n *arrays. The background corrected abundance ratios of sample to reference are obtained for each probe on each array and properly normalized. There are several unique normalization methods proposed for protein microarray data, and the comparison of them are presented by Hamelinck [14].

An analysis of variance (ANOVA) model can be used to partition the sources of variation of the normalized abundance data. The ANOVA model for each protein is

(4) $$Y_{ijkl}=\mu +T_{j}+S_{k(j)}+\delta_{ijk}+\epsilon_{ijkl},$$

where *Y_ijkl _*represents the protein abundance ratio between sample and reference of replicate *l *for sample *k *within group *j *in experiment *i*, *μ *represents the overall mean of the expression ratios, *T_j _*represents the fixed effects of group *j *with constraint ∑*_j _T_j _*= 0, *S_k_*_(*j*)_ represents the random effects of sample *k *within group *j *with mean 0 and variance $\sigma_{Sj}^{2}$, *δ_ijk _*represents the normally distributed random between-experiment effect of experiment *i *for sample *k *in group *j *with mean 0 and variance $\sigma_{\delta }^{2}$, *ϵ_ijkl _*represents the normally distributed random error with mean 0 and variance $\sigma_{\epsilon }^{2}$.

The total of the between-array variation $\sigma_{\delta }^{2}$ and the within-array variation $\sigma_{\epsilon }^{2}$ represents the variation due to random error. Inconsistent proteins inflate both the between-array and within-array variation. By least-squares estimation of the ANOVA model (4), the estimation of $\sigma_{\delta }^{2}+\sigma_{\epsilon }^{2}$ is obtained for each protein and used for classification via a novel semi-nonparametic mixture model approach.

### Semi-nonparametric mixture model

The total of the between-array variation, $\sigma_{\delta }^{2}$, and the within-array variation, $\sigma_{\epsilon }^{2}$, represents the variation due to random error in the ANOVA model (4) and can be estimated for each protein. To select functionally consistent proteins, we assume that all spotted proteins on the arrays represent both functionally consistent and functionally inconsistent proteins with certain proportions that are not too small to be negligible. By modelling the collection of consistency statistics ($\hat{\sigma_{\delta }^{2}}+\hat{\sigma_{\epsilon }^{2}}$) from each protein, using a mixture distribution, it is possible to estimate the consistent or inconsistent status for every protein that is represented on the array. Biologically and technically, consistent proteins are very reliable and are able to generate reproducible measurements between experiments. Because the proposed consistency statistic captures the differences in both consistent and inconsistent proteins, it should be smaller for consistent proteins simply because they have less variation (i.e., are reliable and reproducible) than the inconsistent proteins. Furthermore, statistically, when fitting a two-component mixture model and estimating two components simultaneously, the components have to be identifiable. Therefore, we assume that the mean of the statistics for consistent proteins is smaller than the mean of the statistics for inconsistent proteins, and that the statistics from the same class will be aggregated. For this application, defining a selection criterion is equivalent to finding the classification rule between two classes.

A mixture model with semi-nonparametric densities is proposed, and the Expectation-maximization (EM) quasi-Newton algorithm [34,35] is employed to estimate the parameters. Inferences are then drawn from the estimated mixture model.

Consider the generalized setting where *z*_1_, *z*_2_, ⋯, *z_m _*represent *m *consistency statistics ($\hat{\sigma_{\delta }^{2}}+\hat{\sigma_{\epsilon }^{2}}$), and *f*(*z|θ*) represents their density function. Under the assumption of a two-component mixture, the density *f*(*z|θ*) is equal to a weighted sum as follows

(5) $$f(z|\theta_{0},\theta_{1},\lambda_{0},\lambda_{1})=\lambda_{0}f_{0}(z|\theta_{0})+\lambda_{1}f_{1}(z|\theta_{1}),$$

where *θ*_0 _and *θ*_1 _are the parameters for two densities, *f*_0_(*z|θ*_0_) is the density of the ${z^{'}}_{i}\text{s}$ that are the statistics for functionally consistent proteins, *f*_1_(*z|θ*_1_) is the density of the ${z^{'}}_{i}\text{s}$ that are the statistics for functionally inconsistent proteins, *λ*_0 _is the proportion of the functionally consistent proteins, *λ*_1 _is the proportion of the functionally inconsistent proteins, and the sum of *λ*_0 _and *λ*_1 _is 1. For a mixture model in (5), an order between the means of the two components is assumed. Specifically, let *μ*_0 _and *μ*_1 _represents the means of two components respectively, and assume *μ*_0 _*≤ μ*_1_.

We assume that the density *f*_0_(*z|θ*_0_) and *f*_1_(*z|θ*_1_) belong to a class of semi-nonparametric (SNP) density used by Gallant and Nychka [36]. This smooth class of densities can be represented by a truncated Hermite series expansion, and contain densities that can be skewed, thin or heavy tailed, and multi-modal. The density is represented by

(6) $$f_{i}(z|\theta_{i})={\left[\sum_{j=0}^{K}a_{j}{\left(\frac{z-u_{i}}{v_{i}}\right)}^{j}\right]}^{2}\frac{1}{v_{i}}\phi \left(\frac{z-u_{i}}{v_{i}}\right),$$

where *i *= 0, 1, *K *represents a tuning parameter that is nonnegative, *ϕ*(·) represents a standard normal density.

In order to have *f_i_*(*z|θ_i_*) as a density, a restriction is imposed

(7) $$E{\left[\sum_{j=0}^{K}a_{j}{\left(\frac{z-u_{i}}{v_{i}}\right)}^{j}\right]}^{2}=1,$$

where *z *is a normally distributed random variable. When *K *= 0, *a*_0 _has to be 1 so that *f_i_*(*z|θ_i_*) is exactly the standard normal density. Fortunately, there exists a transformation of coefficients to satisfy the above restriction when *K *is larger than 0. For *K *= 1, the transformation is represented by

(8) $$\begin{array}{l} a_{0}=sin(\phi ),\\ a_{1}=cos(\phi ). \end{array}$$

In this case, *θ_i _*= (*ϕ, u_i_, v_i_*), where *i *= 0, 1. For *K *= 2, the transformation is denoted by

(9) $$\begin{array}{c} a_{0}=sin(\phi_{1})-\frac{cos(\phi_{1})cos(\phi_{2})}{\sqrt{2}},\\ a_{1}=cos(\phi_{1})sin(\phi_{2}),\\ a_{2}=\frac{cos(\phi_{1})cos(\phi_{2})}{\sqrt{2}}. \end{array}$$

Here *θ_i _*= (*ϕ*_1_, *ϕ*_2_, *u_i_, v_i_*), where *i *= 0, 1.

The latent variable *R_i _*takes value 0 if protein *i *is functionally consistent, or 1 otherwise. The likelihood of the complete data (*Z, R*) is

(10) $$L=\prod_{i=1}^{m}{\left[\lambda_{0}f_{0}(z_{i}|\theta_{0})\right]}^{1-R_{i}}{\left[\lambda_{1}f_{1}(z_{i}|\theta_{1})\right]}^{R_{i}}.$$

The log-likelihood is then obtained as follows

(11) logL=∑i=1m[(1−Ri)logλ0+(1−Ri)log f0(zi|θ0)+Rilogλ1+Rilogf1(zi|θ1)].

Based on the log-likelihood (11), maximization techniques can be employed to find the estimates of model parameters, and then classification methods can be implemented based on the estimates of the mixture model.

### EM-algorithm

To estimate the parameters in the log likelihood function (11)the EM-algorithm [34] is employed. There are two steps in the EM-algorithm. In the E-step, the conditional expectation given the data is calculated for missing values *R_i_*

(12) $$\begin{array}{c} E(R_{i})=P(R_{i}=1|z_{i})\\ =\frac{\lambda_{1}f_{1}(z_{i}|\theta_{1})}{\lambda_{0}f_{0}(z_{i}|\theta_{0})+\lambda_{1}f_{1}(z_{i}|\theta_{1})}. \end{array}$$

After substituting in the expectations of missing values, the log likelihood in (11) is maximized (the M-step) by a gradient algorithm that is accelerated by a quasi-Newton method [35]. Given initial values of the parameters, the EM-algorithm iterates between the E-step and the M-step until a convergence criterion is met or until a maximum iteration number is reached.

In the M-step at the (*n *+ 1)*^th ^*iteration, the two parts in the log-likelihood function can be represented by

(13) $$\begin{array}{l} Q_{0}(\theta_{0}|\theta_{0}^{n})=\sum_{i=1}^{n}\left[(1-{\hat{R}}_{i})log\lambda_{0}+(1-{\hat{R}}_{i})log\;f_{0}(z|\theta_{0})\right],\\ Q_{1}(\theta_{1}|\theta_{1}^{n})=\sum_{i=1}^{n}\left[{\hat{R}}_{i}log\lambda_{1}+{\hat{R}}_{i}log\;f_{1}(z|\theta_{1})\right], \end{array}$$

where $\theta_{0}^{n}$ and $\theta_{1}^{n}$ are the estimated parameters in *n^th ^*step. The EM-gradient algorithm [35] updates the parameters as follows,

(14) $$\begin{array}{l} \theta_{0}^{n+1}=\theta_{0}^{n}-{\left(d^{2}Q_{0}(\theta_{0}^{n}|\theta_{0}^{n})-B_{0}^{n}\right)}^{-1}d^{1}Q_{0}(\theta_{0}^{n}|\theta_{0}^{n}),\\ \theta_{1}^{n+1}=\theta_{1}^{n}-{\left(d^{2}Q_{1}(\theta_{1}^{n}|\theta_{1}^{n})-B_{1}^{n}\right)}^{-1}d^{1}Q_{1}(\theta_{1}^{n}|\theta_{1}^{n}), \end{array}$$

where *d*^1 ^represents the first partial derivatives with respect to the parameters, and *d*^2 ^represents the second partial derivatives with respect to the parameters. $B_{0}^{n}$ and $B_{1}^{n}$ are updated in each iteration by applying Davidon's [37] update

(15) $$B_{i}^{n}=B_{i}^{n-1}+a_{i}^{n}c_{i}^{n}{\left(c_{i}^{n}\right)}^{'},$$

where *i *= 0, 1, constant $a_{i}^{n}$ and vector $c_{i}^{n}$ are defined as

(16) $$\begin{array}{l} a_{i}^{n}=\frac{1}{{\left(g_{i}^{n}-B_{i}^{n-1}s_{i}^{n}\right)}^{'}s_{i}^{n}},\\ c_{i}^{n}=g_{i}^{n}-B_{i}^{n-1}s_{i}^{n}, \end{array}$$

with

(17) $$\begin{array}{l} s_{i}^{n}=\theta_{i}^{n-1}-\theta_{i}^{n},\\ g_{i}^{n}=d^{1}Q(\theta_{i}^{n-1}|\theta_{i}^{n})-d^{1}Q(\theta_{i}^{n-1}|\theta_{i}^{n-1}). \end{array}$$

### Determining the number of mixture components

Before applying the two-component mixture model to classify proteins (as consistent or inconsistent), we need to test that the number of components is compatible with the two component mixture model, and that the components can be identified.

Let *g *denote the number of mixture components. The hypothesis to test is

(18) $$H_{0}:g=1\;\text{vs}.\;H_{\alpha }:g=2.$$

Ledwina [38] introduced the idea of a data-driven test for Neyman's smooth test of fit. Here the idea is generalized to the likelihood ratio test of mixture components. The likelihood ratio test statistic is defined as

(19) $$-2log\lambda =-2log\frac{L_{\hat{\theta_{0}}}}{L_{\hat{\theta_{\alpha }}}},$$

where ${\hat{\theta }}_{0}$ and ${\hat{\theta }}_{\alpha }$ represent the estimated parameters under the null and alternative hypothesis, respectively. ${\hat{\theta }}_{0}$ and ${\hat{\theta }}_{\alpha }$ are obtained by choosing the best model via model selection when the two-component mixture model is fit to the data. A bootstrap method is performed to approximate the null distribution of -2*logλ *[39], and to provide a significance threshold for the likelihood ratio test statistic. Specifically, when estimating the null distribution of the likelihood ratio test statistics, we first bootstrap 500 data sets from the estimated distribution under the null hypothesis, and then perform a likelihood ratio test (19) for each simulated data set.

If the test statistic is significant, the two-component mixture model is suitable to fit the data in order to select functionally consistent proteins. Failure to reject the null hypothesis (18) indicates that consistent and inconsistent proteins are not separable, or that there is only one type of protein on the array. For this situation, specific chemical validation techniques have to be employed in order to provide additional consistency information.

### Model selection

For the density in Equation (6), the tuning parameter *K *can be set equal to 0, 1, or 2. To balance the size of parameters and the suitability of the model fit, information criteria are applied to choose the mixture representation that fits the data best. Akaike's Information Criterion (AIC) [28], Schwartz Bayesian Information Criterion (BIC) [29] and Hannan-Quinn Criterion (HQ) [30] are applied, and they all share a penalized log-likelihood in the form of

(20) −2logL+C(N)p,

where *logL *is the log-likelihood, *p *is the number of free parameters in the model, and *C*(*N*) is a function of sample size *N*. AIC requires *C*(*N*) equals constant 2, BIC takes *C*(*N*) = *logN*, and HQ has *C*(*N*) = 2*loglogN*.

### Classification rule and error rate control

Given the estimation of the semi-nonparametric mixture model (5) parameters the posterior probability of protein *i *being functionally inconsistent is calculated by

(21) $$P(R_{i}=1|z_{i})=\frac{{\hat{\lambda }}_{1}\hat{f_{1}}(z_{i}|\theta_{1})}{{\hat{\lambda }}_{0}\hat{f_{0}}(z_{i}|\theta_{0})+{\hat{\lambda }}_{1}\hat{f_{1}}(z_{i}|\theta_{1})},$$

where, ${\hat{\lambda }}_{0},{\hat{\lambda }}_{1}{\hat{f}}_{0}(z_{i}|\theta_{0})$, and ${\hat{f}}_{1}(z_{i}|\theta_{1})$ are the estimates of *λ*_0_, *λ*_1_, *f*_0_(*z_i_|θ*_0_), and *f*_1_(*z_i_|θ*_1_), respectively. The posterior probability of protein *i *being functionally consistent is then obtained by

(22) $$\begin{array}{ccc} P(R_{i}=0|z_{i}) & = & 1-P(R_{i}=1|z_{i}). \end{array}$$

The classification rule that specifies protein *i *as functionally inconsistent protein is defined as

(23) $$P(R_{i}=1|z_{i})\geq c^{*},$$

where c* is the critical value. The selection of the critical value c* is determined by evaluating the estimated false discovery rate (FDR) in Equation (27) and the estimated false non-discovery rate (FNR) in Equation (28). As in Newton *et al*. [40], FDR is estimated by

(24) $$\hat{FDR}=\frac{\sum_{i=1}^{m}P(R_{i}=0|z_{i})\delta_{i}}{\sum_{i=1}^{m}\delta_{i}}.$$

Similarly, FNR is estimated by

(25) $$\hat{FNR}=\frac{\sum_{i=1}^{m}P(R_{i}=1|z_{i})(1-\delta_{i})}{\sum_{i=1}^{m}\left(1-\delta_{i}\right)}.$$

The indicator *δ_i _*is used for declaring protein *i *as functionally inconsistent protein by the classification rule (23) for the specific critical value c*. For any specific protein microarray experiment, the misclassification penalty can be specified. The critical value is obtained by minimizing the following error:

(26) $$\gamma \hat{FDR}\frac{d}{m}+(1-\gamma )\hat{FNR}\frac{m-d}{m},$$

where *γ *∈ [0, 1] is the penalty for false positive, (1 *- γ*) is the penalty for false negative, and *d *is the number of declared inconsistent proteins by the critical value c*.

### Classification error rates

Suppose there are *m *proteins (of which *m*_0 _proteins are truly consistent) that need to be simultaneously classified as consistent and inconsistent (Table 2). Let *R *(an observable variable) denote the number of classified inconsistent proteins, while *U*, *V *, *S*, *T *are unobservable variables. Similar to the false discovery rate (FDR) [41] and false non-discovery rate [42] proposed for multiple testing problems, the misallocation error rates: false discovery rate (FDR) and false non-discovery rate (FNR) are defined as follows. False discovery rate [41], the expected proportion of falsely classified inconsistent proteins among all classified inconsistent proteins, can be represented by

**Table 2:** Classification outcomes: consistent and inconsistent proteins.

	Classified as consistent	Classified as inconsistent	Total
Consistent	*U*	*V*	*m*_0_
Inconsistent	*T*	*S*	*m - m*_0_

Total	*m - R*	*R*	*m*

The total number of proteins is denoted by *m*. *m*_0 _is the number of consistent proteins. *R *is the total number of classified inconsistent proteins. *U, V, T, S *are the corresponding number of classification results.

(27) $$E(Q)=E(\frac{V}{R}|R>0)P(R>0).$$

False non-discovery rate [42], the proportion of falsely classified consistent proteins among all classified consistent proteins, can be represented by

(28) $$E(N)=E(\frac{T}{m-R}|m-R>0)P(m-R>0).$$

## Authors' contributions

RWD initiated the interest in developing statistical methods for protein microarray data, coordinated the research, and finalized the manuscript. LY developed the method, conducted the analysis and the simulations, and drafted the original manuscript. Both authors read and approved the final manuscript.
